# Information Extraction and Summarization for Neurovascular Consultations with GPT-4o: A Clinical Case Study

**DOI:** 10.1007/s00062-025-01538-z

**Published:** 2025-07-31

**Authors:** Ashraya Kumar Indrakanti, Julian Elias Heierle, Hannah Münger, Alma Teresa Koch, Philippe Kaiser, Michael Bach, Jens Fiehler, Ioannis Tsogkas, Raphael Guzman, Matthias Anthony Mutke, Marios Psychogios

**Affiliations:** 1 Department of Diagnostic and Interventional Neuroradiology, Clinic of Radiology and Nuclear Medicine, University Hospital Basel, Petersgraben 4, 4031 Basel, Switzerland; 2 Clinic of Radiology and Nuclear Medicine, University Hospital Basel, Petersgraben 4, 4031 Basel, Switzerland; 3 Department of Neuroradiology, University Medical Center Hamburg-Eppendorf, Hamburg, Germany; 4 Department of Neurosurgery, University Hospital Basel, Petersgraben 4, 4031 Basel, Switzerland

**Keywords:** Outpatient reporting, LLM-assisted medical documentation, Neurovascular consultation

## Abstract

**Purpose:**

In outpatient settings, extensive patient records must frequently be reviewed under time constraints, making efficient extraction and summarization of key clinical information essential. Large language models (LLMs) are potentially useful for this task but require validation for clinical reliability. This study assesses OpenAI’s GPT-4o for generating structured summaries to assist in neurovascular consultation preparation, aiming to increase efficiency by automating critical data extraction.

**Methods:**

A prospective study was conducted from May to August 2024 at a tertiary care hospital, involving a total of 70 patients. Structured summaries were generated by GPT-4o using a predefined template. Extracted data were categorized into aneurysm-specific details, imaging summaries, and patient-specific clinical factors. Accuracy and completeness were assessed by clinicians, with performance measured using precision, recall, specificity, and accuracy.

**Results:**

High accuracy (≥ 0.96) was measured across most categories. In aneurysm-and patient-specific data, extraction performance varied based on stability over time. Aneurysm location and other stable details were extracted consistently, while changes in aneurysm size and medication lists showed variations. In rare cases, aneurysm details were misattributed to a different aneurysm within the same patient. Imaging summaries were generally concise and clinically useful, though their effectiveness declined when summarizing multiple prior studies.

**Conclusion:**

Neurovascular patient data was effectively structured by GPT-4o, demonstrating high accuracy with minimal errors. While occasional misattributions like outdated information were observed, reliable citation of sources facilitated easy verification. These findings support integrating LLM-generated summaries into neurovascular consultations, with further optimization needed for temporal data tracking and on-premise implementation to address privacy concerns.

**Supplementary Information:**

The online version of this article (10.1007/s00062-025-01538-z) contains supplementary material, which is available to authorized users.

## Introduction

The emergence of large language models (LLMs) has significantly influenced a wide range of industries, transforming processes that traditionally relied on extensive human labor. Healthcare, with its vast body of textual information, stands to benefit tremendously from advanced natural language processing capabilities. However, patient care demands exceptional standards of accuracy, reliability, and trustworthiness. Consequently, any implementation of LLMs in clinical workflows must be meticulously validated before introduction. Recent efforts have explored the integration of LLMs in various parts of the healthcare pipeline. Examples are generating report summaries for information consolidation [1], documentation generation [2], and chatbot implementation for interaction with patients [3].

Another potentially effective application of LLMs in healthcare could be in outpatient settings. In such environments, physicians must often navigate extensive volumes of text to retrieve relevant information and construct a coherent overview of a patient’s medical history, prior to meeting the patient. This process can be both time-consuming and cognitively demanding, reducing the available time for direct patient interaction and clinical reasoning.

In our tertiary care university hospital, a specialized weekly consultation is held for patients with neurovascular diseases, largely focused on intracranial aneurysms and, to a lesser extent, other neurovascular diseases such as arteriovenous fistulas or idiopathic intracranial hypertension. Preparing for these consultations often requires reviewing extensive patient histories, including imaging reports, previous procedures, and clinical summaries from various information systems.

The objective of our study was to automate the extraction of structured and semantically rich information from the textual corpus of patients’ neurovascular records by leveraging GPT-4o by OpenAI, a state-of-the-art LLM. The goal was to generate a summary based on a template that clinicians themselves had developed, ensuring alignment with their existing workflows and cognitive models. The summary was tailored to capture essential features of intracranial aneurysms, relevant imaging findings and relevant patient details. We hypothesized that GPT-4o would identify and include all clinically relevant information in its summaries with high precision and completeness.

## Materials and Methods

This prospective study received an ethics waiver from the local institutional Review Board under project-ID Req-2024-01532. All patient-related text was fully anonymized automatically with a subsequent manual check prior to use.

### Study Design and Data Accession

**Fig. 1:**
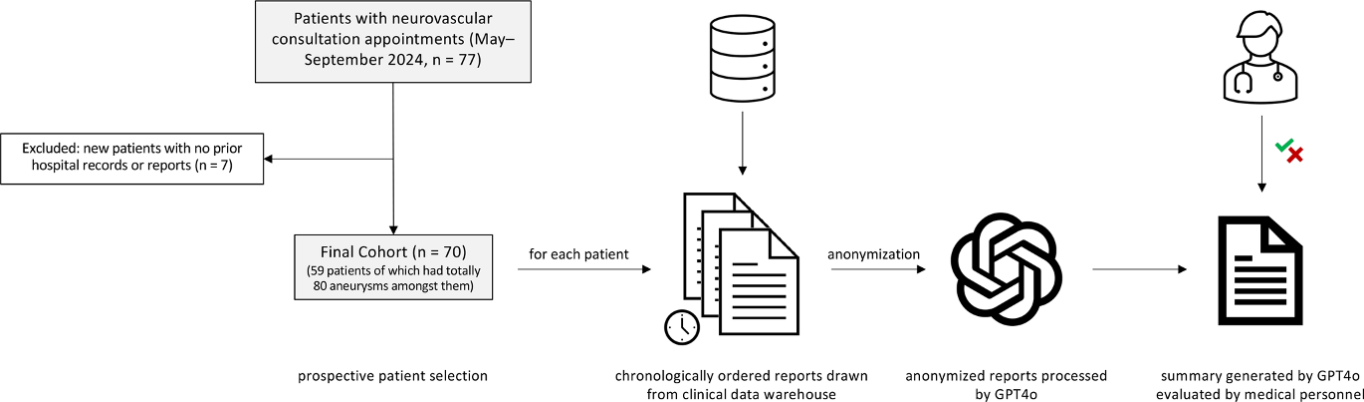
Flowchart of Patients and workflow. All patients with neurovascular consultation appointments between May and September 2024 with prior records stored in the hospital database were included in the study. For every patient, all cranial imaging reports, neurovascular treatment reports and neurovascular consultation reports were drawn from the hospital clinical data warehouse and chronologically concatenated into one continuous text. After anonymization, this text was processed by the GPT-4o model and yielded a summary, which was then evaluated by clinicians

The study was conducted prospectively from May to August 2024 at our university hospital. During this period, patient history summaries generated by an LLM were prepared in advance for all neurovascular consultations (see Fig. 1 for an overview of data collection and processing). Clinicians, as part of their routine preparation for these consultations, reviewed these summaries and assessed their correctness and completeness. Following this preliminary evaluation, further detailed analyses were conducted to derive quantitative metrics of the LLM’s performance (see Data Evaluation below).

We accessed all relevant patient reports stored as text in our hospital’s clinical data warehouse (developed in-house) via SQL database queries. The data warehouse integrates patient data from multiple clinical information systems within the hospital, enabling streamlined access for scientific and clinical evaluation. The reports used for creating the summaries included cranial imaging reports, neurovascular consultation notes, and neurovascular treatment records. Patient-identifying information was removed after the data retrieval process.

### Data Processing

We used GPT-4o (version: gpt-4o-2024-05-13, the latest version available at the commencement of the study) as the LLM to generate the summaries. For generating summaries (and for any output in general), the LLM requires two components as input: a system prompt and a user prompt. In our context, the system prompt provided explicit instructions to the LLM on the task to be performed, while the user prompt contained the information which was to be processed. The specific system prompt utilized in this study is detailed in the supplementary material. The temperature hyperparameter was set to 0. Temperature controls how freely the model can vary its wording; setting it to 0 greatly reduces variability in the output between identical input. We chose this setting to maximize performance consistency. All data and its evaluation were in German.

To ensure the generation of consistent and accurate outputs, five separate neurovascular patient cases were retrospectively selected for generating and refining the system prompts prior to the beginning of the study. The system prompt (provided in the supplement) implements well-established prompting techniques [4], like role allocation (“You are a trainee interventional neuroradiologist specializing in intracranial aneurysms …”), context explanation (“… need to prepare notes for a patient consultation …”), showing input-output examples (few-shot learning), and urging the model to self-check its output. The type of examples within the prompt, especially with varying formulations and representations, notably improved the output quality.

For each patient, the user prompt consisted of chronologically connected text. This text included previous cranial imaging reports, neurovascular consultation records, and neurovascular treatment summaries. All patient identifying information was systematically removed before inputting the data into the LLM. Apart from variations in the user prompt due to patient-specific data, all other parameters were kept constant across all generated summaries.

For every consultation, the LLM generated a summary following a predefined template developed by clinicians. This template mirrored the format routinely used by clinicians to prepare for consultations. The template was structured into three sections. The first section (Section 1) contained the following aneurysm-specific structured information: aneurysm size, date and context of first diagnosis, rupture state, localization, shape, wall condition, thrombus state, scores (PHASES [5], ELAPSS [6], UIATS [7]), and treatment details. The second section (Section 2) provided free-text summaries of all previous cranial imaging reports, focusing on the imaging indication, key findings and emphasizing aneurysm details if present. The third section (Section 3) contained patient-specific structured information on relevant clinical details: neurovascular risk factors (smoking, diabetes, hypertension, hyperlipidemia, alcohol consumption) and medication regimen, including dosage and frequency.

Section 1 was evaluated only for patients with documented intracranial aneurysms, and the summary was evaluated separately for each aneurysm (i.e. “aneurysm-wise”), since some patients had multiple aneurysms, and the information varied from aneurysm to aneurysm. Sections 2 and 3 were aneurysm-independent and were therefore assessed for all patients for each patient (i.e. “patient-wise”). Each piece of information documented in the summaries was accompanied by a citation of the source (identified by the date and imaging modality, treatment or consultation). The LLM was specifically instructed to replicate the exact phrasing and wording present in the original reports to ensure fidelity.

### Data Evaluation

The evaluation of Sections 1 and 3 was focused on the extraction of structured information from patient records. These sections were used to assess the LLM’s ability to retrieve and summarize specific data points accurately. Since the task is very similar to a detection problem, the performance of the LLM was evaluated based on the following measures: True Positives (TP) were instances of information present in the original records and correctly documented in the summary; True Negatives (TN) referred to information absent in the original record and correctly identified as absent in the summary; False Positives (FP) involved incorrect information documented in the summary; and False Negatives (FN) represented relevant information missed by the LLM. In cases where the documented information was partially correct but lacked key details, it was classified as “True incomplete”.

Secondary metrics were calculated from the definitions above: Precision (also known as Positive Predictive Value, PPV; TP / (TP + FP)), Recall (Sensitivity; TP / (TP + FN)), Negative Predictive Value (NPV; TN / (TN + FN)), Specificity (= TN / (TN + FP)), and Accuracy (= (TP + TN) / (TP + TN + FP + FN)) were calculated based on these values. For the purposes of these calculations, true but incomplete information was categorized as False Negatives, as missing components imply an absence of full information in the summary.

Additional information was also documented and included elements in the summary with missing sources, incorrect sources, or non-connected information (information included in a section of the template to which it is not relevant or applicable). These were considered independently from the primary performance measures.

For Section 2, which involved the summarization of cranial imaging reports, a clinician marked all information in the summary as either relevant or irrelevant for the consultation at hand. Since the output of this section was less structured than that of Sections 1 and 3, A “usefulness quotient”, defined as the number of useful words divided by the whole number of words in section, was then calculated for each summary.

## Results

Among the 70 patients included in the analysis, 59 were diagnosed with intracranial aneurysms, with a total of 80 aneurysms amongst them. The remaining patients had arteriovenous fistulae (8 patients), consultations post stent implantation (2 patients) and consultation post stroke (1 patient). Of the 59 patients with aneurysms, 11 patients had two aneurysms, two patients had three aneurysms, and two patients had four aneurysms.

### Section 1

**Fig. 2:**
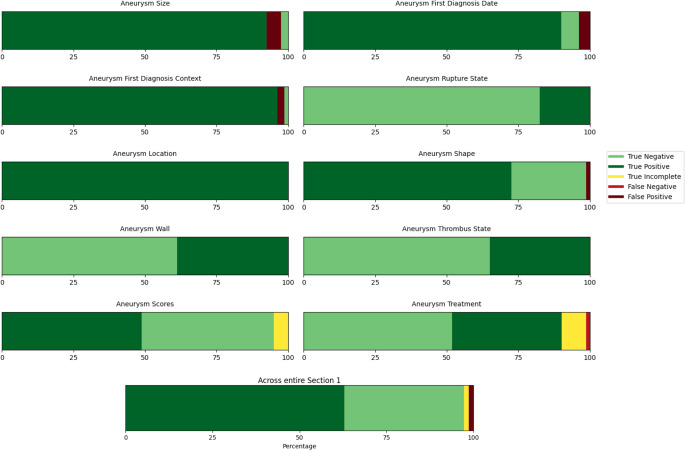
Evaluation results of Section 1, for each subcategory and across the whole section

Figure 2 and Table 1 show the results in graphical and tabular form, respectively. Across all evaluated secondary metrics, performance levels were 0.96 or higher.

**Table 1:** Evaluation of Section 1 across all subcategories. Includes primary measure counts (True Positives, True Negatives, False Positives, False Negatives), secondary metrics quotients (Precision, Recall, Specificity, NPV (Negative Predictive Value), Accuracy) and further additional information counts (Instances of missing sources, wrong sources and non-connected information)

	Size	First Diagnosis Date	First Diagnosis Context	Rupture State	Location	Shape	Wall	Thrombus State	Scores	Treatment	Total
True Positives	74	72	77	14	80	58	31	28	39	31	*504*
False Positives	4	3	2	0	0	1	0	0	0	0	*10*
True Negatives	2	5	1	66	0	21	49	52	37	42	*275*
False Negatives	0	0	0	0	0	0	0	0	0	1	*1*
True Incomplete	0	0	0	0	0	0	0	0	4	7	*11*
Precision (PPV)	0.95	0.96	0.97	1	1	0.98	1	1	1	1	*0.98*
Recall (Sensitivity)	1	1	1	1	1	1	1	1	0.91	0.79	*0.98*
Specificity	0.33	0.63	0.33	1	NA	0.95	1	1	1	1	*0.96*
NPV	1	1	1	1	1	1	1	1	0.90	0.84	*0.96*
Accuracy	0.95	0.96	0.98	1	1	0.99	1	1	0.95	0.90	*0.97*
Missing Source	0	2	1	0	1	1	0	0	1	0	*6*
Wrong Source	0	0	0	0	0	1	0	0	0	0	*1*
Non-connected Information	0	0	0	1	0	0	2	0	0	0	*3*

Recall and NPV were especially high across all categories due to the very low occurrence of FN. Exceptions to this are the Scores and Treatment categories, where there were multiple True Incomplete elements. The model sometimes failed to include all relevant treatment details for patients with multiple treated aneurysms, including the treatment of only one aneurysm instead of all of them. Similarly, aneurysm scores like UIATS, which contain multiple numeric components, were not always captured in full. Nonetheless, the absolute error counts remained low, and in most instances of error, the extracted data were largely correct but partially incomplete. Since the True Incomplete elements were added to FN for the metrics, this led to lower values in those categories.

Features such as Rupture State, Location, Shape and Wall were consistently correctly recognized (all metrics ≥ 0.95), likely because these attributes remained stable over time and served as strong “anchors” for the model to link the textual data to specific aneurysms. Across these four categories, there was only one error. In contrast, data points that varied across time or required careful chronological interpretation, such as First Diagnosis Date or Aneurysm Size, occasionally led to misattributions. These errors typically stemmed from selecting information from suboptimal source reports rather than the most recent ones. Although these mistakes were rare, they were straightforward to detect and rectify given the LLM’s practice of citing source documents.

Specificity was sometimes quite low (see categories Size, First Diagnosis Date and First Diagnosis Context: between 0.33 and 0.63). This is simply because the number of Negatives for those categories was very low, leading the FP to overrun the TN. This metric is suboptimal for categories with a very small number of total Negatives.

### Section 2

**Fig. 3:**
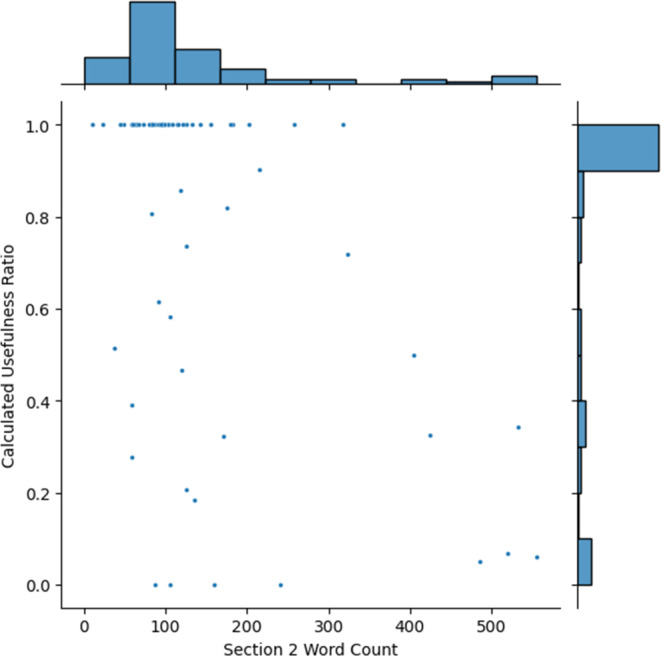
Scatterplot of Section 2: Relationship between the length of section 2 and the usefulness ratio of the text. A histogram for each axis shows respective univariate distribution

Section 2 evaluated how the model summarized previous cranial imaging findings. Across all summaries, there were no imaging studies which were missed by the LLM. There were no hallucinations (erroneous data generation) provided in the summary. There were three cases where the summary of a report was incomplete (missing pieces of information in those three cases were treatment type, aneurysm sizes and imaging indication), and one wrong information (The size of the aneurysm was referenced from another study involving the same patient, as indicated in the summary). None of the missing information was deemed critical by the reviewing clinicians for the upcoming consultation. Median length of section 2 was 98.5 words (IQR: 84.25 words), and median usefulness quotient was 1.0 (mean: 0.77, IQR: 0.49). Although the results were generally satisfactory, a trend emerged: as the length of section 2 grew longer, the usefulness quotient of these summaries decreased (see Fig. 3). For the clinicians, summaries of the most recent one or two imaging studies were often considered important and clinically relevant to the upcoming consultation. Summaries of older imaging studies were often considered unimportant due to the lower clinical relevance to the upcoming consultation. Patients with a higher number of previous cranial imaging reports also had a higher number of older imaging studies which tended to be considered irrelevant, leading to lower usefulness quotients. The evaluation suggested that while GPT-4o performed well at distilling the core findings from key imaging reports, the value of including exhaustive historical imaging data might diminish as the chronological gap widens.

### Section 3

**Fig. 4:**
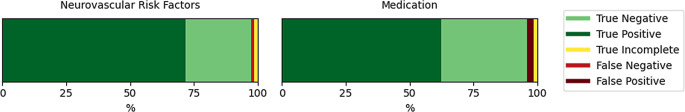
Evaluation results of Section 3, for each subcategory

Section 3, addressing neurovascular risk factors and current medications of patients, matched the strong performance observed in Section 1 (see Fig. 4; Table 2). For the neurovascular risk factors, the LLM was almost always accurate in the summary. Only one case of a missed smoking history was reported. In terms of medications, GPT-4o struggled occasionally with interpreting notes about medication cessation. In two cases, if a medication was actively listed as “to be stopped” in a report, the model still included it in the current medication list, failing to interpret that the patient was no longer taking it. Apart from these errors, GPT-4o demonstrated a high degree of accuracy in summarizing information related to medication and neurovascular risk factors.

**Table 2:** Evaluation of Section 3 across all subcategories. Includes primary measure counts (True Positives, True Negatives, False Positives, False Negatives), secondary metrics quotients (Precision, Recall, Specificity, NPV (Negative Predictive Value), Accuracy) and further additional information counts (Instances of missing sources, wrong sources and non-connected information)

	nvRF	Medication
True Positives	55	45
False Positives	0	2
True Negatives	19	24
False Negatives	1	0
True Incomplete	1	1
Precision (PPV)	1	0.96
Recall (Sensitivity)	0.96	0.98
Specificity	1	0.92
NPV	0.90	0.96
Accuracy	0.97	0.96
Missing Source	3	3
Wrong Source	0	0
Non-connected Information	0	0

### Further Details

**Fig. 5:**
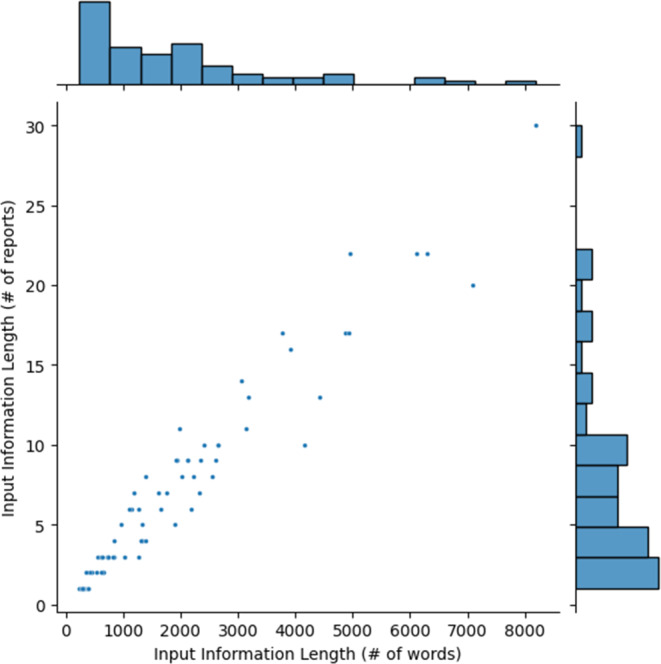
Scatterplot of total LLM-generated summary: Relationship between the input data word count and the number of reports of the input data. A histogram for each axis shows respective univariate distribution

Figure 5 graphically illustrates the length of the input data, both in number of words and number of reports. Median number of reports in the input was 6 (IQR: 6.75 reports), and median word count of the input was 1359 (IQR: 1878 words).

There were 9 cases of Missing Sources in total, seven of which occurred in one single report. There was one case of a wrong source noted in the summary, in a case where the subheading of the cited report “Anamnese” (“Patient History”) was noted instead of the title of the report “MRI vom …” (“MRI of …”). So, apart from these two cases, the sources extracted from the LLM were extremely reliable.

All non-aneurysm patients were correctly identified by the LLM to not have an aneurysm. No substantial hallucinations (erroneous data generation) occurred regarding the existence of aneurysms. However, in one case, an extra aneurysm was documented, resulting in five aneurysms being reported for the patient instead of the actual four. In this specific instance, the patient had two aneurysms located in the A2/3 segment of the anterior cerebral artery in both hemispheres. In some reports, one of these aneurysms was inconsistently described as being in the pericallosal artery (another name for the A3 segment), while other reports described its location as the A2/3 segment. Although inconsistent reporting occurred in other cases, such as varying descriptions like “paraophthalmic” versus “supraophthalmic,” in this case, the LLM was unable to infer that the A3 segment is equivalent to the pericallosal artery and invented a new aneurysm in response to the inconsistent documentation.

## Discussion

This study demonstrates that GPT-4o can reliably extract structured information from unstructured, extensive and heterogeneous neurovascular medical reports. While the use of LLMs has been investigated in certain summarization tasks [1, 8], the application of LLMs for structured information extraction and patient history summarization in clinical outpatient settings is an underexplored area, and this study highlights their potential to fill this gap effectively.

The model’s performance is exemplary across most evaluated metrics, with scores consistently exceeding 0.95. It demonstrated robust accuracy in extracting structured information from clinical reports, capturing essential details with high fidelity. Its ability to identify relevant information, maintain context, and accurately cite sources highlights its potential as a reliable tool for supporting clinical workflows.

One core strength of GPT-4o is its ability to process input text remarkably well without requiring domain-specific fine-tuning [9]. The raw, non-curated clinical reports used in this study contained local terminology and figure of speech, variable reporting styles, and occasionally contradictory information. Despite these challenges, the model produced summaries that allowed clinicians to enter consultations with a concise and verified understanding of their patients’ conditions. The simplicity of the prompting strategy offers clinical end-users a relatively low barrier to entry. With basic techniques of prompt engineering (e.g. role assignment, few-shot, self-evaluation, all used in this setting), a physician or medical staff member can instruct the model to produce results that follow a given template, use the same wording as in the reports, and reference the sources accordingly. Unlike approaches that require elaborate post-processing or strict data formatting (like forcing a JSON (JavaScript Object Notation) output, which is a lightweight, human- and machine-readable text format for structured data, e.g. {“size”:“8 mm”, “shape”:“saccular”}), this method relies on the model’s intrinsic understanding of textual structure.

Another important aspect is the model’s efficiency. Producing results rarely took longer than twenty seconds, demonstrating its potential to save considerable time in outpatient settings. The general processing speed, along with the model’s reliable sourcing capabilities enable quick verification of the summarized information. This suggests that, with further optimization, LLMs could significantly streamline the preparation of patient histories for consultations. One possibility is to build a User Interface on top of an LLM-powered application providing easy access to the original sources of each piece of information, to speed up verification. This could help clinicians invest more time in patient interaction and shared decision-making rather than navigating long and complex medical histories, especially considering that the median word count of the input was more than 1300 words, equivalent to about three full pages of text. If integrated properly, summary generation would take virtually no additional time. Given ten consultations per week, each requiring ten minutes of manual preparation, automation could save 100 min weekly per clinician. At an estimated hourly rate of 200 CHF for specialized personnel, this translates to annual savings of roughly 17,000 CHF per clinician, with significant cost reductions at the hospital level.

Despite its strong performance, errors were observed, particularly in extracting temporally complex information and allocating attributes to the correct entity in situations involving multiple aneurysms or evolving clinical details. For conditions such as intracranial aneurysms, attributes like size, wall condition and medication details often change over time. In such cases, the LLM sometimes struggled to accurately track this dynamic information and pick the correct details (e.g. failing to recognize when a drug was stopped). Additionally, the model faltered when dealing with inconsistent naming conventions, such as variations in describing the pericallosal artery. In one instance, these inconsistencies led to the erroneous documentation of an additional aneurysm. These challenges underscore the inherent difficulty of extracting and reconciling temporally and contextually complex information, which could be a general issue in other clinical scenarios involving multiple pathologies or dynamic changes.

Data privacy and regulatory issues must be carefully considered in the application of LLMs in clinical settings. In this study, prior to the data processing via GPT-4o, all data was anonymized, and all patient identifiers were removed. However, the same pipeline of using external servers for data processing cannot be directly applied to clinical use due to privacy concerns regarding data transmission and confidentiality. Clinical settings may require entirely local, on-premise solutions to meet stringent data protection requirements. Although GPT-4o may not be directly suitable for clinical use due to this reason, the results shown in this study can act as a reference point to evaluate the effectiveness of locally hosted or fine-tuned alternatives.

Our study focused on a specific use case: structured information extraction and patient history summarization in outpatient settings, utilizing sources such as radiology, consultation, and neurointerventional reports. While these sources were sufficient for this particular clinical context, other specialties or more complex patient histories might benefit from integrating additional data sources such as laboratory results, pathology reports, or primary care records. The virtues and challenges identified in this study can, to a certain extent, be extrapolated to other outpatient settings with similar requirements in the context of medical information summarization. The next steps could involve comparing the model’s extraction capabilities against those of trained human reviewers or incorporating active learning loops where clinicians correct LLM outputs, thereby refining future performance.

In conclusion, GPT-4o demonstrated exceptional performance in medical text summarization and structured information extraction, consistently achieving high accuracy across diverse and complex clinical data. Its ability to handle real-world, non-curated reports without domain-specific fine-tuning underscores its robustness and adaptability. While errors occurred in handling temporally complex data or multiple entities, these were minor and easily traceable due to reliable sourcing. While not directly implementable in clinical settings due to data privacy concerns, GPT-4o provides a valuable reference point for evaluating local models and highlights the transformative potential of LLMs in streamlining clinical workflows and enhancing patient care across medical specialties.

## Supplementary Information

The Supplement contains the system prompt used for extracting and organising the information from the input reports.
